# MRI in intracavitary brachytherapy planning for cervical cancer malignancy, the pitfalls and complications

**DOI:** 10.1186/1470-7330-15-S1-P36

**Published:** 2015-10-02

**Authors:** G Coniglio, O Roche, C Maciel, A Sahdev

**Affiliations:** 1 St Bartholomew's Hospital, W Smithfield, London, EC1A 7BE, UK

## Learning objectives

- Discuss the role and benefits of MRI in planning for brachytherapy treatment in cervical cancer.

- Discuss the key mechanisms of the applicator and the technical aspects of planning brachytherapy.

- Discuss the role of MRI imaging in recognising the features of appropriate applicator placement and common complications.

- Review key MRI features of radiation-related alterations in the pelvis post treatment and tumour response assessment.

## Contents

- Anatomy of the female pelvic organs on MRI

- Key MRI imaging features of cervical cancer on MRI

- Review the staging of cervical cancer amenable to brachytherapy (FIGO)

- Advantages of brachytherapy versus external beam radiotherapy in treating cervical cancer.

- Technical aspects of applicator selection and positioning in brachytherapy.

- MRI features in assessment of appropriate placement of brachytherapy device, recognition of “organs at risk”.

- Benefits of MRI versus more traditional imaging techniques CT and Radiographs, in brachytherapy planning.

- “Red flag” features of brachytherapy device misplacement in MRI.

- Features of tumour response and target organ assessment.

- Complications of brachytherapy including post radiation fibrosis.

## Conclusion

- Analysis of MRI findings at the time of brachytherapy with the applicator is essential in the assessment of gross tumour volume, clinical target volumes and patho-anatomical structures.

- T2-weighted MR images minimise potential misinterpretation due to partial volume effects, which improves depiction of tumour in parametria, vaginal fornices, and cervix.

